# Daniel Osiris

**Published:** 1907-03

**Authors:** 


Daniel Osiris, the philanthropist, a wealthy Greek, died at Paris, February 4, 1907. He purchased Malmaison, the château a few miles from Paris, which was the residence of the Empress Josephine from 1798 to 1815, renovated it and, in December, 1900, presented it to the French people for use as a Napoleonic museum. He replaced some of the former fittings and furniture, and employed artists to obliterate the traces of pillage left by the Prussians and the Communists.

In 1899 M. Osiris presented to the Instiute of France a sum representing an annual income of about $6,500 for a triennial prize of $20,000, open to all countries, for the most remarkable work or discovery of general interest, especially in the fields of surgery and medicine.

